# Genetic Variation of the Serotonin 2a Receptor Affects Hippocampal Novelty Processing in Humans

**DOI:** 10.1371/journal.pone.0015984

**Published:** 2011-01-18

**Authors:** Björn H. Schott, Constanze I. Seidenbecher, Sylvia Richter, Torsten Wüstenberg, Grazyna Debska-Vielhaber, Heike Schubert, Hans-Jochen Heinze, Alan Richardson-Klavehn, Emrah Düzel

**Affiliations:** 1 Leibniz-Institute for Neurobiology, Magdeburg, Germany; 2 Department of Neurology, University Hospital of Magdeburg, Magdeburg, Germany; 3 Department of Psychiatry, Campus Mitte, Charité University Hospital, Berlin, Germany; 4 German Center for Neurodegenerative Disease (DZNE), Magdeburg, Germany; 5 Institute for Cognitive Neurology and Dementia Research, University Hospital of Magdeburg, Magdeburg, Germany; 6 Institute of Cognitive Neuroscience, University College London, London, United Kingdom; Georgia Health Sciences University, United States of America

## Abstract

Serotonin (5-hydroxytryptamine, 5-HT) is an important neuromodulator in learning and memory processes. A functional genetic polymorphism of the 5-HT 2a receptor (5-HTR2a His452Tyr), which leads to blunted intracellular signaling, has previously been associated with explicit memory performance in several independent cohorts, but the underlying neural mechanisms are thus far unclear. The human hippocampus plays a critical role in memory, particularly in the detection and encoding of novel information. Here we investigated the relationship of 5-HTR2a His452Tyr and hippocampal novelty processing in 41 young, healthy subjects using functional magnetic resonance imaging (fMRI). Participants performed a novelty/familiarity task with complex scene stimuli, which was followed by a delayed recognition memory test 24 hours later. Compared to His homozygotes, Tyr carriers exhibited a diminished hippocampal response to novel stimuli and a higher tendency to judge novel stimuli as familiar during delayed recognition. Across the cohort, the false alarm rate during delayed recognition correlated negatively with the hippocampal novelty response. Our results suggest that previously reported effects of 5-HTR2a on explicit memory performance may, at least in part, be mediated by alterations of hippocampal novelty processing.

## Introduction

Episodic memory [1], the ability to encode, store and recall single, typically autobiographical, events in their spatial and temporal context, is critically dependent on the hippocampus and adjacent medial temporal lobe (MTL) structures [2], [3], an observation supported by functional neuroimaging experiments [1], [4], [5] that have provided evidence for prefrontal-hippocampus interactions during successful encoding of stimuli into episodic memory. One important function of the hippocampus in episodic memory is the detection and encoding of novel information [6], [7], [8], [9].

Long-term encoding of novel stimuli in the hippocampus has been linked to co-activation of glutamatergic and neuromodulatory monoaminergic receptors. Serotonergic projections from the medial septal and median raphe nuclei to the hippocampus are thought to modulate hippocampal memory processes [10], [11], and, genetic investigations have yielded a replicated association of genetic variations of the serotonin receptor 5-HTR2a with human memory. Particularly the 5-HTR2a His452Tyr polymorphism (dbSNP: rs6314), which influences the intracellular signaling cascade of the receptor [12], [13], has been demonstrated to affect episodic memory, with lower performance in carriers of the rare Tyr variant [14], [15], [16]. The neural correlates of this effect at brain systems level, however, are yet unclear. 5-HTR2a is expressed in the human hippocampus and prefrontal cortex (PFC), and expression in these brain structures decreases with age [17], [18], which is mirrored by a reduced influence of His452Tyr on memory performance in the elderly [15].

Based on the well-established role of the hippocampus in novelty processing and the replicated association of 5-HTR2a with hippocampus-dependent memory, we hypothesized that the polymorphism might affect hippocampal processing of novel stimuli. This hypothesis was addressed using functional magnetic resonance imaging (fMRI) in 5-HTR2a His homozygotes and Tyr carriers. Participants performed a visual novelty/familiarity task with photographs of complex scenes (see Bunzeck and Düzel, 2006, for a similar task) that was followed by a delayed recognition test 24 hours later.

## Materials and Methods

### Ethics statement

All study participants gave written informed consent to participate, in accordance with the Declaration of Helsinki, and the study was carried out in accordance with the guidelines of the local ethics committee.

### Participants

41 young (age range 19–28, including 17 Tyr carriers; genotyping protocol available upon request), right-handed native speakers of German (19 female) participated in the study. All had no history of neurological or psychiatric illness and normal T1-weighted MR images.

### Paradigm

**Figure 1:**
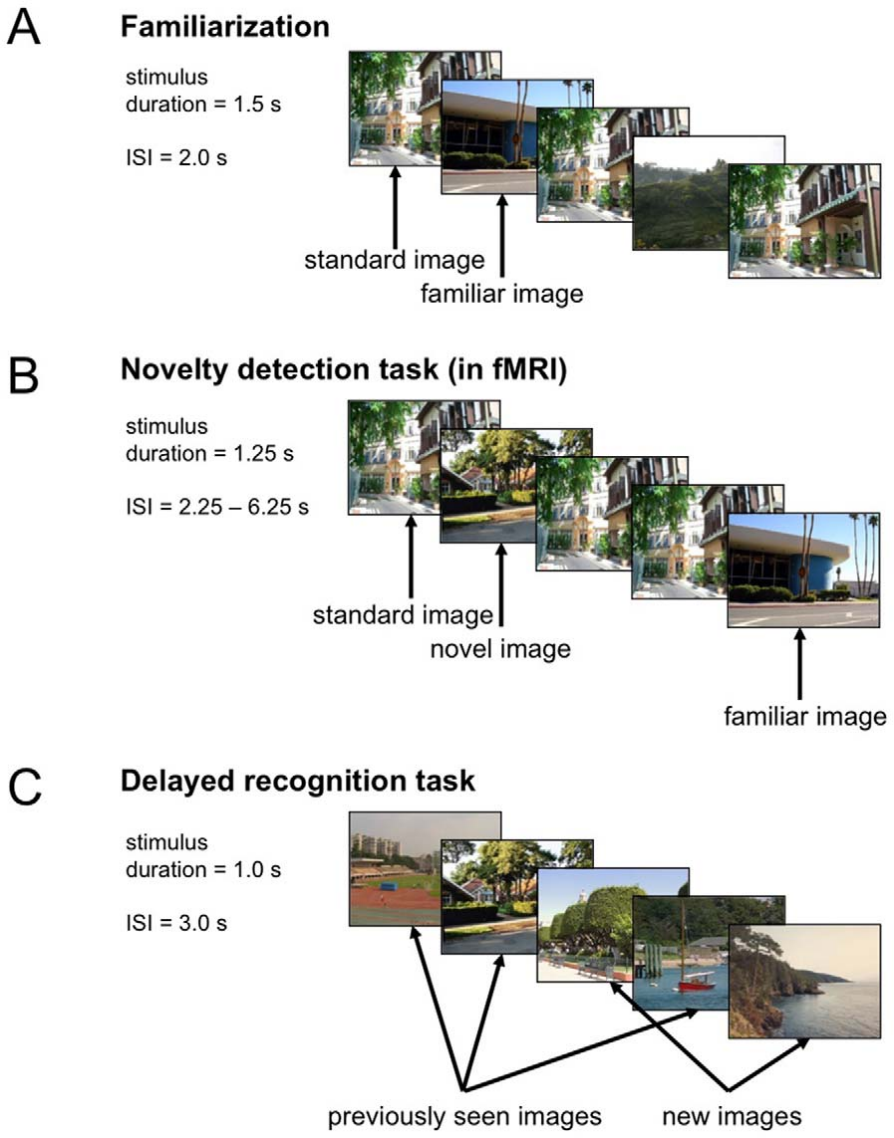
Experimental paradigm. A: During the initial familiarization phase, before the actual fMRI experiment, seven pictures were presented repeatedly. A standard image was presented 60 times, and six familiar targets were repeated 10 times in a pseudo-random order. B: During the fMRI experiment, novel and familiar target stimuli (photographs of outdoor scenes) were presented, randomly intermixed with a standard image. 240 photographs were presented, including 120 repetitions of the standard image, 60 familiar targets (each repeated 10 times), and 60 novel targets. C: During delayed recognition (24 hours after scanning), the novel targets from the fMRI experiment were presented randomly intermixed with previously unpresented images.

Figure 1 displays the experimental setup of the task. Before entering the MR tomograph, participants performed a familiarization phase, during which they viewed a total of seven photographs of outdoor scenes on a computer screen. A standard image was repeated 60 times, and six target images were repeated 10 times in a pseudo-random Latin square order (Figure 1A).

The actual experiment consisted of a single fMRI scanning session. During the fMRI experiment, novel and familiar target stimuli were presented, randomly intermixed with a standard image. Photographs of outdoor scenes were presented in a pseudo-randomized order (stimulus duration = 1.25 s), with an interstimulus interval (ISI) jittered between 2.25 s and 6.25 s with a near-exponential distribution, to optimize estimation of the BOLD response [19]. A total of 240 photographs were presented, including 120 repetitions of the standard image, 60 familiar targets (the six target pictures from the familiarization phase, each repeated 10 times), and 60 novel targets (see Figure 1B). Participants were instructed to respond via button press whether the target images were familiar or novel, but ignore the standard image. The order of images was newly randomized across participants, as was the subset of novel targets, which consisted of 120 images, the other half being used as distractors in the delayed recognition phase (see below).

24 hours after the novelty/familiarity task, participants performed a delayed recognition test (Figure 1C). The 60 novel targets from the fMRI experiment were presented again, randomly intermixed with 60 previously unseen photographs. Participants were instructed to respond via mouse button whether or not they recognized the pictures from the previous day. False positive responses were explicitly discouraged.

### MRI acquisition

MR images were acquired on a GE 1.5T Signa MRI system (General Electric) using a standard head coil. 450 T2*-weighted echo-planar images [EPIs; TR = 2.0 s; TE = 35 ms; 23 axial slices (64×64); voxel size = 3.13×3.13×5 mm (4 mm slice thickness+1 mm gap)] were acquired (odds first, from bottom to top). Six volumes were acquired at the beginning of each run to allow for magnetic field stabilization. A co-planar proton density (PD)-weighted MR image was acquired before the functional session and used for optimized normalization (see below).

### Data processing and analysis

Data was analyzed using Statistical Parametric Mapping (SPM8b, Wellcome Trust Center for Neuroimaging, London, UK). EPIs were corrected for acquisition delay, realigned, normalized using the parameters determined from segmentation of the co-planar PD image [voxel size: 3×3×3 mm] and smoothed [Gaussian kernel; FWHM = 8×8×8 mm]. A high pass filter of 128 s was applied to the data.

**Figure 2:**
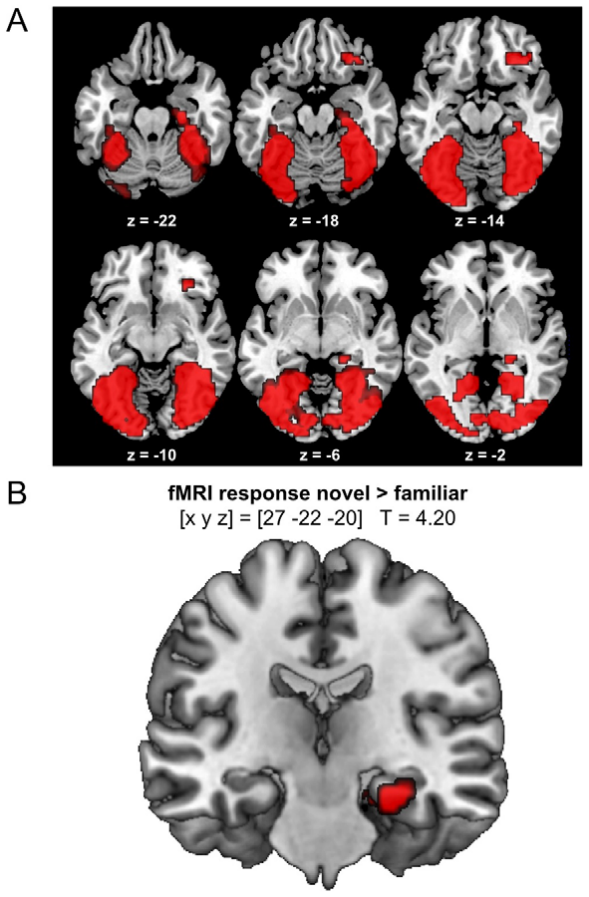
Neural correlates of novelty processing. A: The presentation of novel versus familiar target stimuli was associated with activation of a distributed network within the ventral visual stream, including secondary visual, fusiform, and parahippocampal cortices. B: There was a significant negative activation of the hippocampus during presentation of novel relative to familiar target pictures (p<.05, small-volume FWE-corrected).

Statistical analysis was performed in a two-stage Mixed Effects model. In the first stage, neural activity was modeled by a delta function at stimulus onset. The blood oxygen level-dependent (BOLD) event-related responses were modeled by convolving these delta functions with a canonical hemodynamic response function (HRF). The resulting time courses were downsampled for each scan to the regressors of interest (novel and familiar target stimuli, standard picture) included in a General Linear Model (GLM). Covariates were modeled for the conditions of interest (novel and familiar target stimuli, standard picture), the six rigid-body movement parameters determined from realignment, and a single constant representing the mean over scans. Parameters of the GLM for each covariate were estimated by restricted maximum likelihood (ReML) fit. Second level random effects analyses were computed over the single subjects' contrasts. To assess the interaction between novelty and 5-HTR2a genotype, we first assessed genotype-specific variation of the novelty responses by computing a genotype by novelty (novel vs. familiar targets) interaction model, followed by a planned between-group comparison of novel vs. familiar T-contrasts. Because of our hypothesis regarding the well-established role of the hippocampus in novelty processing, which was replicated in the present study cohort (Figure 2), we assumed that genotype-mediated differences in novelty processing would likely be observed in the hippocampus and conducted a region of interest (ROI) analysis, using an anatomically defined ROI of the hippocampus (CA regions, subiculum; SPM Anatomy Toolbox [20]). The significance threshold was set to p<.05, small-volume-corrected for family-wise error (FWE). Peak activations (fitted and adjusted responses) of clusters with significant between-group differences were submitted to confidence interval estimation using Bootstrap resampling and the percentile-t method [21]. Only activation differences with non-overlapping confidence intervals for His homozygotes and Tyr carriers were considered reliable.

## Results

### 5-HTR2a His452 genotype and recognition performance

**Figure 3:**
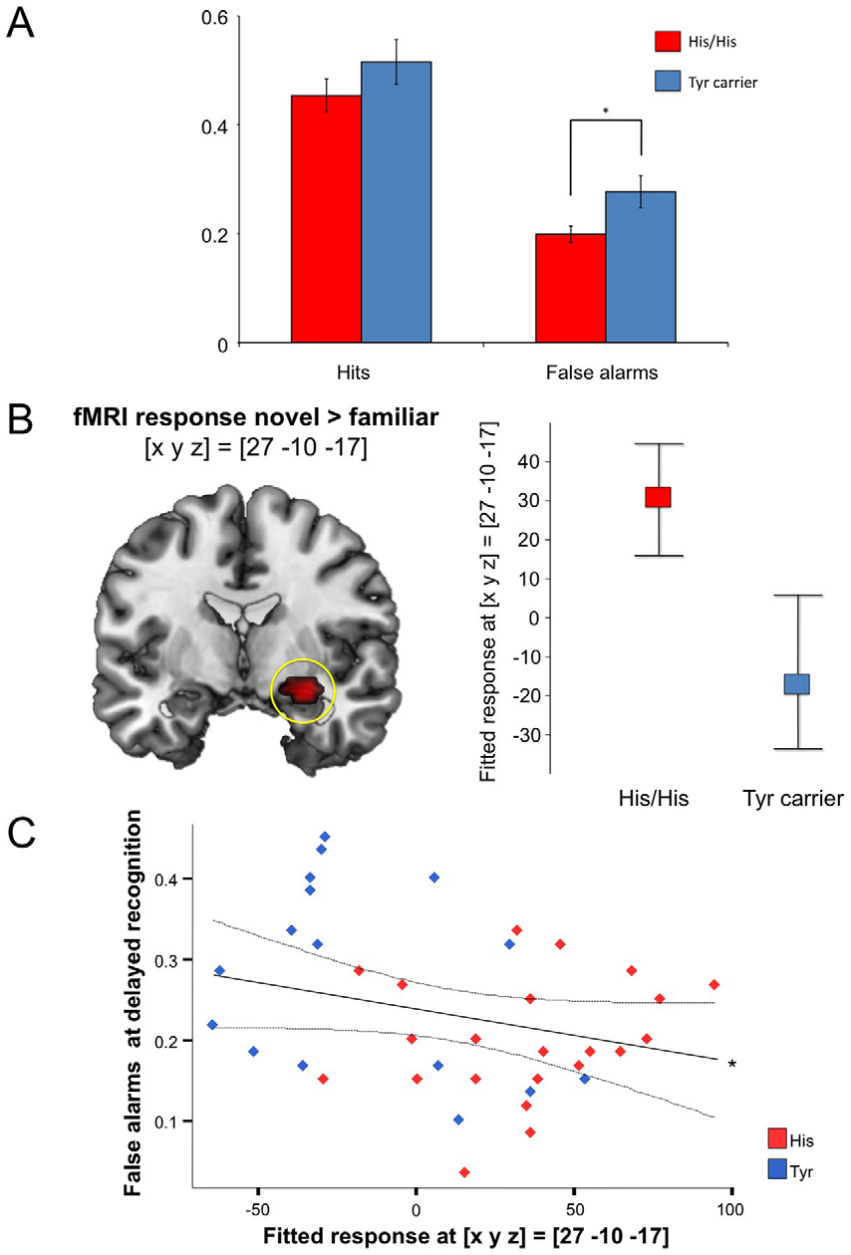
The hippocampal novelty response and its modulation by the 5-HTR2a His452Tyr genotype. A: While there was no significant genotype-related difference in hit rate (correctly recognized old pictures) during delayed recognition, Tyr carriers made significantly more false alarms, i.e. “old”-like responses to new stimuli (T = −2.564; p = .015). B: His homozygotes showed relatively increased activation of the right anterior hippocampus when compared to Tyr carriers. Plots depict mean activations (fitted and adjusted response), separated by genotype, +/− confidence intervals obtained from Bootstrap resampling; p<.05, FWE-corrected for the ROI volume. C: There was a significant negative correlation of the hippocampal novelty response and the false alarm rate during delayed recognition across the study cohort. *r = .278; p<.043, one-tailed.

The average percentages of correctly recognized familiar items (hits) and novel items classified as familiar (false alarms) during the novelty task and during delayed recognition are displayed in Table 1, separated by 5-HTR2a genotype. [*Note:* Delayed recognition results were not available from two participants (one His/His, one Tyr carrier). Behavioral results from the recognition phase are therefore based on 39 participants]. Participants of both groups judged more previously seen items as familiar relative to unseen items, but Tyr carriers made significantly more false alarms during delayed recognition [T_39_ = −2.564; p = .015, two-tailed] (Figure 3A). Reaction times (RTs) during delayed recognition showed a significant condition by genotype interaction, with Tyr carriers showing shorter RTs only for items judged old (hits, and particularly false alarms), confirming their tendency to judge stimuli as old [F_1,37_ = 3.959; p = .022, one-way ANOVA for repeated measures with genotype as between-subjects factor, and Greenhouse-Geisser correction for non-sphericity].

**Table 1:** Behavioral results.

Novelty detection task	*His/His*	*Tyr carrier*	
**% hits**	**.992**+/−.013	**.985**+/−.029	n.s.
**% false alarms**	**.003**+/−.008	**.038**+/−.120	n.s.
RT hits	**775**+/−86	**787**+/−125	n.s.
RT correct rejections	**813**+/−96	**838**+/−124	n.s.

Delayed recognition	*His/His*	*Tyr carrier*	
**% hits**	**.454**+/−.148	**.516**+/−.164	n.s.
**% false alarms**	**.199**+/−.074	**.277**+/−.118	T = −2.564; p = .015*
RT hits	**945**+/−170	**913**+/−105	n.s.
RT misses	**955**+/−214	**956**+/−133	n.s.
RT correct rejections	**934**+/−199	**948**+/−130	n.s.
RT false alarms	**964**+/−197	**893**+/−127	n.s.

Relative proportions of hits (correctly recognized pictures) are given. [*Note:* Delayed recognition results were not available from two participants (one His/His, one Tyr carrier)]. Behavioral results from the recognition phase are therefore based on 39 participants]. RT: reaction time (msec); n.s.: not significant. All data are means +/− standard deviations.

### 5-HTR2a His452 genotype influence on fMRI correlates of novelty processing

#### Brain activity related to novelty processing

Irrespective of genotype, novel target pictures elicited increased activation of an extensive ventral visual network, including the fusiform and parahippocampal gyrus when compared to familiar pictures (Figure 2A). We also observed bilateral hippocampal activation for novel versus familiar target pictures (p<.05, small-volume FWE-corrected; Figure 2B), compatible with previous observations [22].

#### Effects of 5-HTR2a His452Tyr on hippocampal novelty processing

A ROI analysis using an anatomically defined ROI of the hippocampal CA regions and subiculum (derived from the SPM Anatomy Toolbox; Eickhoff et al., 2005) revealed a significant genotype-dependent between-group difference in the right anterior hippocampus. Carriers of the Tyr allele showed significantly reduced right anterior hippocampal activation during presentation of novel relative to familiar target images, as compared to His homozygous participants (p<.05, FWE-corrected for the ROI volume; see Figure 3B). To reduce the risk of incidental between-group differences, peak activation differences were submitted to Bootstrap-based confidence interval estimation [21]. Confidence intervals for the fitted and adjusted hemodynamic responses in the anterior hippocampus did not overlap between His homozygotes and Tyr carriers (Figure 2, top right).

To test whether the hippocampal novelty response was related to behavioral novelty processing, we computed Pearson's correlation coefficients between the hippocampal hemodynamic response to novel vs. familiar stimuli and the hit and false alarm rates during delayed recognition. While the hit rate did not correlate significantly with the hippocampal novelty response, we observed a negative correlation between this response and the delayed recognition false alarm rate [r = .278; p<.043, one-tailed], suggesting an inverse relationship between hippocampal response to novel stimuli and a tendency to mistakenly judge novel stimuli as familiar (Figure 3C) [*Note:* the correlation was negative in both genotype groups, but reached significance only across the entire cohort].

## Discussion

While behavioral studies using several different memory tasks have provided converging evidence for an influence of 5-HTR2a His452Tyr on human memory performance, the underlying neural correlates have thus far been unclear. Here we show a relationship between 5-HTR2a His452Tyr and hippocampal novelty processing, suggesting that the polymorphism might affect the hippocampus-dependent encoding of novel stimuli.

In young, healthy volunteers, the hippocampal neural response to novel stimuli varied as a function of 5-HTR2a His452Tyr. This observation is compatible with previously reported lower performance of Tyr carriers in hippocampus-dependent memory tasks, irrespective of performance in other cognitive tasks. Effects of 5-HTR2a His452Tyr on novelty processing were observed almost exclusively in the hippocampus, while no genotype-dependent activation differences were found in the PFC, where the receptor is also expressed at high levels [17], [18]. This might reflect a preferential effect of the polymorphism on hippocampal as compared to neocortical function, which would be in line with the previously described relatively specific effects of the polymorphism on explicit memory processes [14], [15]. It should be noted, though, that the task employed here was specifically designed to elicit stable hippocampal novelty responses and was not associated with prominent prefrontal activations in our entire cohort.

Previous studies investigating neuromodulatory influence on novelty processing have mostly focused on dopamine [7], [9]. Hippocampal activation, possibly in response to novelty, can lead to dopamine release in the hippocampus via a positive feedback loop with the substantia nigra / ventral tegmental area [7], and dopamine, in turn, is necessary to maintain and stabilize hippocampal long-term potentiation (LTP), a putative synaptic correlate of long-term memory formation. A comparable role for serotonin has been described [23], and pharmacological studies have linked serotonergic neurotransmission to memory performance [24]. Ca^2+^ influx is critical for the expression of hippocampal LTP, and the Tyr allele exerts a destabilizing effect on the Phospholipase C (PLC) signaling cascade downstream of the 5-HTR2a receptor, leading to a decreased calcium response [12], [13]. It should be noted that serotonergic projections in the hippocampus terminate, to a large extent, on GABAergic interneurons [25] and therefore, serotonin may also modulate novelty processing at the level of hippocampal network dynamics [26].

An alternative – or additional – explanation our observations would be that modulation of memory function by 5-HTR2a His452Tyr might result from developmental effects. Indeed, morphometric analyses have shown altered MTL white matter microstructure and reduced hippocampal volume in Tyr carriers [27], suggesting a possible effect of His452Tyr on MTL plasticity or development.

### Conclusions

Taken together, the results of the present study show that previously reported effects of the His452Tyr functional variation of the 5-HT receptor 2a on human memory performance might, at least in part, be mediated by decreased recruitment of the hippocampus during novelty processing. Our results suggest that, in addition to dopamine, serotonin warrants further investigation as a putative neuromodulator of hippocampus-dependent processing of novel information. Given the widely replicated studies linking hippocampal dysfunction and schizophrenia and suspected role of 5-HTR2a polymorphisms, including His452Tyr, in risk for schizophrenia and response to atypical antipsychotics [28], we further suggest that the differential roles of serotonin and dopamine in psychosis-related memory dysfunction should be subject to future research.
